# Predicting the Adult Clinical and Academic Outcomes in Boys With ADHD: A 7- to 10-Year Follow-Up Study in China

**DOI:** 10.3389/fped.2021.634633

**Published:** 2021-08-02

**Authors:** Yanling Ren, Xinyu Fang, Hui Fang, Gaofeng Pang, Jing Cai, Suhong Wang, Xiaoyan Ke

**Affiliations:** ^1^Affiliated Nanjing Brain Hospital, Nanjing Medical University, Nanjing, China; ^2^The Third Affiliated Hospital of Soochow University, Changzhou, China

**Keywords:** ADHD, predictor, IQ, family factors, academic outcome, clinical outcome

## Abstract

**Background:** Attention-deficit/hyperactivity disorder (ADHD) often persists into adulthood and causes adverse effects on social functioning. The present study aimed to widely investigate the predictors, particularly childhood intelligence quotient (IQ) and family environment factors, on adult clinical and academic outcomes in boys with ADHD.

**Methods:** A total of 101 boys with ADHD in a Chinese Han ADHD cohort were followed up 7–10 years later. Baseline ADHD symptoms were evaluated using the parent version of the ADHD Rating Scale-IV (ADHD-RS-IV) and the Chinese version of the Conners' Parent Rating Scale-Revised (CPRS-48). The intelligence of the child was tested by the China-Wechsler Intelligence Scale for Children (C-WISC), and family function was assessed by the Family Environment Scale-Chinese Edition (FES-CV). Adult ADHD persistence was defined using DSM-IV criteria for ADHD, and academic outcome fell into two categories: higher academic level group (studying in senior middle school or above) and lower academic level group (studying in vocational secondary schools or below).

**Results:** Stepwise multiple logistic regression analysis revealed that the father's character, impulsive–hyperactive index as measured by the CPRS-48, and intellectual–cultural index as measured by the FES-CV independently predicted clinical outcomes in adults, with an AUC of 0.770 (*p* < 0.001, 95% CI = 0.678–0.863). The corresponding sensitivity and specificity were 0.743 and 0.727, respectively. The father's education level, family economic level, and verbal IQ (VIQ) on the C-WISC independently predicted adult academic outcomes, with an AUC of 0.870 (*p* < 0.001, 95% CI = 0.796–0.944). The corresponding sensitivity and specificity were 0.813 and 0.783, respectively.

**Conclusion:** Initial ADHD symptom severity and IQ, father's character and education level, and family atmosphere and function affect adult clinical and academic outcomes. Addressing these areas early may help to improve the prognosis of ADHD into adulthood.

## Introduction

Attention-deficit/hyperactivity disorder (ADHD) is one of the most prevalent childhood-onset neurodevelopmental disorders and is characterized by symptoms of hyperactivity, impulsivity, and inattention (1). It affects 3~7% of school-age children (2), especially boys (3). Epidemiological data have documented that 60~80% of children with ADHD continue to show symptoms in adulthood. Generally, children with ADHD are susceptible to cognitive impairments, lower self-esteem, sexual and social problems, and psychiatric comorbidities (4–6). In addition, it has profound impacts on the education, career, and social functions in the adulthood of persistent patients (7). Currently, ADHD has caused a heavy social burden and become a serious public health problem (8, 9). Hence, identifying the factors in childhood that predict the persistence of ADHD and associated impairments in adulthood is important for early detection and targeted intervention as well as the prevention or reduction of long-term negative outcomes.

Many studies have indicated that the childhood intelligence quotient (IQ) may be an important predictor of adult ADHD persistence. A 2-year longitudinal study demonstrated that childhood IQ could predict ADHD persistence (10), which was also replicated in a recent long-term follow-up study (11). In addition, a more recent study focusing on the longest cohort of ADHD participants revealed that childhood IQ was positively associated with educational level, occupational rank, and social and occupational adjustment (12). Interestingly, Roy et al. (13, 14) found a significant association between childhood IQ and academic outcome but not adult ADHD persistence (13, 14). The discrepancy in the previous findings may be attributed to several factors, including age at IQ assessment, sex ratio, and follow-up interval (15, 16). IQ encompasses many aspects of performance and is usually divided into verbal intelligence quotient (VIQ) and performance intelligence quotient (PIQ). Furthermore, evidence supports that VIQ and PIQ vary across children with ADHD (17). VIQ and PIQ may have different impacts on adult clinical and academic outcomes. However, very few studies to date have focused on predicting the long-term prognosis of children with ADHD, distinguishing VIQ and PIQ. Therefore, further studies are needed to clarify which IQ predicts adult clinical and academic outcomes in children with ADHD.

Moving beyond IQ, the predictive indicators of family factors, such as family economic status, character and education level of parents, parental rearing patterns, parental relationship and health status of parents, especially maternal psychopathology and psychosocial adversity, have been reported in previous studies (15, 18–20). Although family factors may not be the key mechanism for the cause of ADHD, they do play a crucial role in the entire process of the condition (21). A recent cohort study found that parental education and family income were significantly associated with adult functional outcomes in ADHD (14), but were not associated with adult ADHD symptom persistence (13). Hence, these two family factors need more investigation. Moreover, dysfunction in family interaction manifests as inconsistent discipline, exposure to trauma, and conflict between parents that may worsen the symptoms of ADHD (15). Several scales were used to assess the degree of discord between the parents and the level of parent–child conflict, such as the Family Environment Scale (FES). The Chinese version of the FES-CV includes 10 dimensions of family environment factors, such as cohesion, expressiveness, conflict, independence, achievement, intellectual–cultural, active–recreational, moral–religious, organization, and control (22). It has become one of the most important and practical tools for assessing family atmosphere and function. A recent study showed that the ADHD group had lower cohesion, expressiveness, independence, achievement, intellectual–cultural, moral–religious, organizational, and control scores but higher conflict scores than the non-ADHD group (23). To our knowledge, no study has explored which family environmental factors predict adult clinical and academic outcomes based on the FES.

Therefore, our present study focused on using the FES-CV to investigate family environmental factors and using the parent version of the ADHD Rating Scale-IV (ADHD-RS-IV), Chinese version of the Conners' Parent Rating Scale-Revised (CPRS-48), and China-Wechsler Intelligence Scale for Children (C-WISC) to evaluate ADHD symptoms and IQ (VIQ and PIQ) in boys with ADHD. Furthermore, we followed this Chinese Han ADHD cohort and aimed to provide a comprehensive investigation of the predictors of adult clinical and academic outcomes. We hypothesized that hostile family environmental factors and poor VIQ or PIQ might have a predictive role on adult ADHD outcome, but VIQ and PIQ may have different effects.

## Methods

### Participants

All participants were recruited from the Child Psychology Outpatient Department of the Third Affiliated Hospital of Soochow University, China from June 2009 to June 2013. Participants were originally included when they met the following criteria: (1) had been unanimously diagnosed with ADHD according to the Diagnostic and Statistical Manual of Mental Disorders, Fourth Edition (DSM-IV) alongside the Kiddie Schedule for Affective Disorders and Schizophrenia (KSADS) by at least two experienced psychiatrists; a third psychiatrist appeared in cases of dissent between the first two psychiatrists; (2) boy aged 6–12 years, Han Chinese; (3) full-scale IQ (FSIQ) >70; and (4) first visit to the hospital and never received drugs for ADHD. Patients were excluded if they were diagnosed with schizophrenia, mood disorder, autism spectrum disorder, or physical and neurological disease.

A total of 154 boys with ADHD completed the evaluation and were originally involved in the study, including 34 (33.66%) ADHD inattentive type (ADHD-I), 2 (1.98%) ADHD hyperactive-impulsive type (ADHD-HI), and 65 (64.36%) ADHD combined type (ADHD-C) subjects at baseline. The researchers did not interfere with the treatment given by the psychiatrist. They were all contacted to participate in the follow-up after they were 18 years of age (ranging from 18.01 to 23.80 years) from June 2019 to June 2020. The procedure was carried out in accordance with the latest version of the Declaration of Helsinki. This study was approved by the local Medical Ethics Committee of the Third Affiliated Hospital of Soochow University and was registered in the China Clinical Trial Registration Center: ChiCTR1800015877. Guardians of all subjects provided signed informed consent prior to the performance of any procedures related to this study.

### Baseline Measures

A self-designed questionnaire was used to collect demographic and clinical data of the participants and their parents, including age at diagnosis, family economic status, parental educational level, child and parental character, maternal health during pregnancy, only child or not, birth and delivery status, and family history. The age at diagnosis, maternal health during pregnancy, and birth and delivery status were recorded based on medical information. Family economic status was defined as the total monthly household income level, measured on a categorical scale of 1–3 (higher level: more than RMB 10,000, middle level: RMB 5,000–10,000, and lower level: less than RMB 5,000) (24). Character was evaluated by the items on the extraversion subscale of the Eysenck Personality Questionnaire (Children's and Adult's Versions). Children and their parents were then classified into Introvert (score below 43.3), Ambivert (score between 43.3 and 56.7), or Extrovert (score higher than 56.7) groups according to the standard Extraversion scores (25).

ADHD symptoms were evaluated using the parent version of the ADHD Rating Scale-IV (ADHD-RS-IV), which is a valid and widely utilized measurement tool used in school-age children with ADHD. The ADHD-RS-IV consists of 18 items corresponding to DSM-IV criteria for ADHD and is based on a four-point scale. Each symptom is scored based on how often it occurs (i.e., if they “never” presented the symptom, it is rated as 0; if “occasionally,” 1; “often,” 2; and “always,” 3). The score yielded by this instrument ranges from 0 (symptoms “never” occur) to 54 (all symptoms “always” occur) (26). The total symptom scores as well as the inattention and hyperactivity–impulsivity subscale scores were used to evaluate the core symptoms of ADHD. The Chinese version has been translated and has good validity and reliability (27).

The Chinese version of the Conners' Parent Rating Scale-Revised (CPRS-48) was also used to evaluate symptoms and behaviors in boys with ADHD (28), which included 48 questions on a four-point Likert scale from 0 (for normal) to 3 (for severe). A higher score represents a more severe behavioral problem. The CPRS-48 uses six subscales to evaluate different behavioral outcomes: conduct problems, learning problems, psychosomatic problems, impulsive–hyperactive, anxiety, and ADHD index. Raw scores for each subscale were converted into sex- and age-adjusted *T*-scores within a mean ± standard deviation (SD) of 50 ± 10. The Chinese version of the CPRS-48 works well in evaluating symptoms and behaviors in children with ADHD, with the homogeneity reliability of Cronbach's α, the correlation of the Spearman-brown split-half, and the retest reliability of the total score being 0.932, 0.900, and 0.594, respectively (28). In the present study, the CPRS-48 was independently completed by guardians according to the instruction manual under the direction of trained investigators.

The intelligence of the child was tested by the China-Wechsler Intelligence Scale for Children (C-WISC), which was revised by Gong and Cai at Hunan Medical University. The C-WISC consists of 11 individual tests that include six verbal tests [Information (I), Comprehend (C), Sorting (S), Arithmetic (A), Vocabulary (V), and Digit symbol (D)] and five performance tests [Picture Completing (PC), Picture Arrangement (PA), Block Pattern (BP), Object Assembly (OA), and Coding (CD)]. Based on individual testing, vocabulary scores (V), procedure scores (P), and full scores (F) were obtained. Furthermore, the VIQ, PIQ, and full intelligence quotient (FIQ) were calculated progressively (29). All evaluations were conducted by experienced professional staff who were well-trained for this project, and repeated assessments revealed that a correlation coefficient of more than 0.8 was maintained.

We used the Family Environment Scale-Chinese Edition (FES-CV) for family environmental factor investigation, which included 90 items on 10 dimensions: cohesion, expressiveness, conflict, independence, achievement, intellectual–cultural, active–recreational, moral–religious, organization, and control. The interpretation of each item on the FES-CV is detailed in the previous literature (22). Each item was answered as “Yes = 1” or “No = 2” by one of the parents who spent more time with family and became involved in more family affairs. The FES-CV has good validity and reliability in Chinese people and works well in evaluating different kinds of families and testing the family relationships and family environment in China (23).

### Follow-Up Assessment and Outcomes

Supplementary Figure 1 shows the process of recruitment and follow-up. A total of 101 subjects agreed to a face-to-face or telephone interview and were included in the final analysis. The average length of follow-up was 9.31 (±1.15) years. The remaining 53 children with ADHD dropped out because they were unable to be contacted. A self-designed questionnaire was used to collect the demographic and clinical data of the participants and their parents, including age, education level, and records of treatment. As all the children with ADHD had completed the 9-year compulsory education at follow-up (finished junior high school at an average age of 15.27 ± 0.30), we divided them into two academic outcome groups: (a) higher academic level group (studying in senior middle school or above) and (b) lower academic level group (studying in vocational secondary schools or below).

The DSM-IV was used for the Diagnostic Interview for ADHD in adults by trained researchers at a follow-up time point, and the clinical outcomes were defined in the following three categories: (a) subjects meeting full DSM-IV criteria for ADHD, (b) subjects meeting partial criteria for ADHD (meeting functional impairment or meeting only part of the diagnostic criteria but falling short of symptom criteria), and (c) subjects not meeting the DSM-IV criteria for ADHD at all. We further divided the adult subjects into two groups: the symptomatic persistence group (a and b) and the symptomatic relief group (c) (30).

### Data Analysis

The Statistical Package for the Social Sciences (SPSS) version 23.0 was used for data analysis. First, we report the demographic data and clinical and academic outcomes of the subjects. Second, Student's *t*-tests (homoscedasticity), *t*′-tests (homoscedasticity uneven), or chi-squared tests were used to compare the differences between groups as appropriate. Bonferroni corrections were used in scale comparisons between groups for multiple tests. Third, the variables that were statistically significant (*p* < 0.05) in the group comparison (the total scale score was not included) were then entered into the stepwise logistic regression analysis (forward selection), with *p*-value criteria of 0.01 and 0.05 for entry and removal, respectively, and clinical or academic outcomes as the dependent variables. Finally, receiver operating characteristic (ROC) curves were used to assess the predictive effect of variables on outcomes. All tests were two-sided, and a *p*-value of 0.05 was used as the threshold for statistical significance.

## Results

### Factors Predicting Adult Clinical Outcomes in Boys With ADHD

#### Characteristics of the Symptomatic Persistence Group and Symptomatic Relief Group at Follow-Up

In the present study, only 35 of 101 (34.65%) boys with ADHD achieved symptomatic relief in early adulthood. There were no significant differences in age at follow-up (19.54 ± 1.08 vs. 19.69 ± 1.51, *t* = 0.575, *p* = 0.566) or follow-up interval (9.18 ± 1.17 vs. 9.56 ± 1.08, *t* = 1.612, *p* = 0.110) between the symptomatic persistence group and the symptomatic relief group. However, our results showed that the symptomatic relief group had a better academic outcome than the symptomatic persistence group (χ^2^ = 4.872, *p* = 0.027). Furthermore, the baseline demographic and clinical information of the follow-up group was similar to that of the drop-out group (all *p* > 0.05), except for the significant difference in the control index on the FES-CV (*t* = 2.141, *p* = 0.034).

#### Comparisons of Baseline Characteristics Between the Symptomatic Persistence Group and Symptomatic Relief Group

Table 1 shows the baseline demographic and clinical characteristics between the symptomatic persistence and symptomatic relief groups. Our results showed no significant differences in age at diagnosis, ADHD subtype, only child or not, mother's character, child's character, parental educational level, family economic level, maternal health during pregnancy, birth and delivery status, family history, and treatment or not between the symptomatic persistence group and the symptomatic relief group (all *p* > 0.05). However, the symptomatic persistence group had a higher incidence of oppositional defiant disorder than the symptomatic relief group (χ^2^ = 4.166, *p* = 0.041). The fathers in the symptomatic persistence group tended to be more introverted than those in the symptomatic relief group (χ^2^ = 8.841, *p* = 0.012). For the rating scale, the symptomatic persistence group had higher subscale scores on impulsive–hyperactive (*t* = 2.226, *p* = 0.028) and ADHD index (*t* = 2.067, *p* = 0.041) on the CPRS-48 and lower subscale scores on intellectual–cultural (*t* = 2.524, *p* = 0.013) and active–recreational index (*t* = 2.235, *p* = 0.028) on the FES-CV compared to the symptomatic relief group. Only the difference on the intellectual–cultural index endured after Bonferroni correction (*p* = 0.026). There were no other differences on the CPRS-48, FES-CV, ADHD-RS-IV, or C-WISC between the two groups at baseline (all *p* > 0.05).

**Table 1:** Comparisons of baseline characteristics between symptomatic persistence group and symptomatic relief group.

	**Persistent group**	**Remittent group**	***t*/*X*^**2**^**	**P**
	***N* = 66**	***N* = 35**		
Age at diagnosis (year)	10.37 ± 1.43	10.13 ± 1.54	0.757	0.451
Education level				
Father	10.89 ± 2.88	12.03 ± 3.59	1.729	0.087
Mother	10.79 ± 3.06	11.40 ± 3.51	0.910	0.365
Family economic (High/Mid/low)	7/57/2	3/31/1	0.111	0.946
Treatment (Yes/No)	36/30	21/14	0.277	0.599
ADHD type (I/HI/C)	20/2/44	14/0/21	1.857	0.395
Character (Introvert/Extrovert/ Ambivert)				
Father	23/27/16	4/14/17	8.841	0.012
Mother	6/41/19	2/21/12	0.571	0.752
Child	17/26/23	8/17/10	0.806	0.668
Only child (Yes/No)	56/10	31/4	0.265	0.606
Maternal health during pregnancy (Yes/No)	42/24	28/7	2.879	0.090
Delivery status (Normal natural labor/Cesarean section/Abnormal natural labor)	32/28/6	16/17/2	0.560	0.756
Birth status (health/Unhealth)	55/11	28/7	0.174	0.677
Oppositional defiant disorder (Yes/No)	11/55	1/34	4.166	0.041
Family history (Yes/No)	21/45	10/25	0.113	0.736
ADHD-RS-IV				
Inattention	16.67 ± 3.25	15.74 ± 2.94	1.403	0.164
Hyperactivity-impulsivity	12.00 ± 4.87	11.34 ± 4.96	0.641	0.523
CPRS-48				
Conduct problems	0.92 ± 0.49	0.74 ± 0.45	1.744	0.084
Learning problems	1.78 ± 0.59	1.56 ± 0.62	1.700	0.092
Psychosomatic problems	0.30 ± 0.32	0.21 ± 0.22	1.550	0.124
Impulsive-hyperactive index	1.38 ± 0.54	1.11 ± 0.60	2.226	0.028
Anxiety	0.51 ± 0.41	0.46 ± 0.45	0.487	0.627
ADHD index	1.31 ± 0.42	1.11 ± 0.50	2.067	0.041
IQ				
VIQ	102.09 ± 14.89	108.23 ± 17.26	1.865	0.065
PIQ	99.14 ± 13.12	99.31 ± 10.82	0.066	0.948
FIQ	100.83 ± 13.09	104.54 ± 11.98	1.395	0.166
FES-CV				
Cohesion	7.25 ± 1.47	7.42 ± 1.57	0.544	0.587
Expressiveness	5.35 ± 1.36	5.59 ± 1.49	0.807	0.422
Conflict	3.50 ± 2.03	3.30 ± 1.84	0.487	0.628
Independence	5.40 ± 1.38	5.36 ± 1.15	0.163	0.871
Achievement	6.48 ± 1.58	6.41 ± 1.85	0.201	0.841
Intellectual-cultural	3.72 ± .1.73	4.66 ± 1.90	2.524	0.013
Active-recreational	3.32 ± 2.11	4.25 ± 1.75	2.235	0.028
Moral-religious	5.20 ± 1.37	4.89 ± 1.60	1.051	0.296
Organization	6.28 ± 1.81	6.04 ± 2.18	0.585	0.560
Control	3.71 ± 1.88	3.50 ± 1.75	0.533	0.595

*Data were presented in Mean ± SD or N*.

#### Risk Factors for Predicting Adult Clinical Outcomes in Boys With ADHD

**Figure 1:**
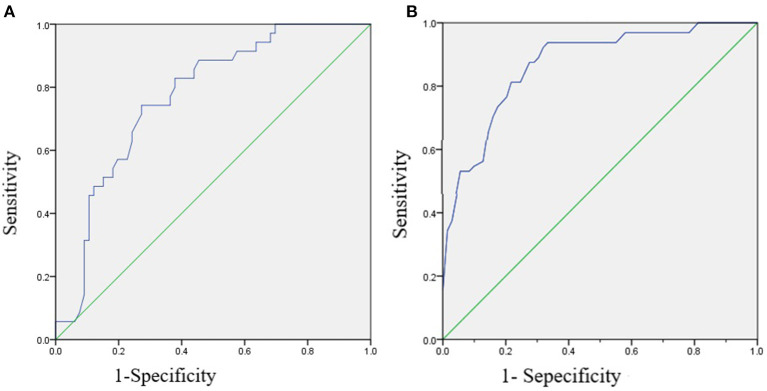
The receiver operating characteristics (ROC) curve of stepwise logistic regression prediction model for adult clinical and academic outcomes in boys with ADHD. **(A)** The ROC curve of stepwise logistic regression prediction model for adult clinical outcome, with the AUC as 0.770 (*p* < 0.001, 95% CI = 0.678–0.863), and the corresponding sensitivity and specificity were 0.743 and 0.727, respectively. **(B)** The ROC curve of stepwise logistic regression prediction model for adult academic achievement, with the AUC as 0.870 (*p* < 0.001, 95% CI = 0.796–0.944), and the corresponding sensitivity and specificity was 0.813 and 0.783, respectively.

With the variables screened by group comparison as independent variables (*p* < 0.05), a stepwise multiple logistic regression analysis was used to determine the independent predictive factors for symptomatic persistence in boys with ADHD. As shown in Table 2, the results revealed that the father's character (Extrovert and Ambivert as dummy variables, Introvert as the reference), impulsive–hyperactive index on the CPRS-48, and intellectual–cultural index on the FES-CV could independently predict clinical outcomes in adults. The results suggest that this model is a good predictor of symptomatic persistence in adulthood, with an AUC of 0.770 (*p* < 0.001, 95% CI = 0.678–0.863). The corresponding sensitivity and specificity were 0.743 and 0.727, respectively (see Figure 1A).

**Table 2:** Results of the stepwise logistic regression analysis: independent predictors for adult clinical outcome in ADHD boy.

						**95% CI for Exp (B)**	
	**B**	**SE**	**Wald**	**Sig**	**Exp. (B)**	**Lower**	**Upper**
Father's character			10.447	0.005			
Father's character (Extrovert)	−2.250	0.704	10.219	0.001	0.105	0.027	0.419
Father's character (Ambivert)	−0.951	0.534	3.177	0.075	0.386	0.136	1.099
Impulsive-hyperactive index	−0.970	0.432	5.033	0.025	0.379	0.162	0.885
Intellectual-cultural index	0.314	0.134	5.448	0.020	1.368	1.052	1.781

*The variable of father's character was set as 2 dummy variables (Extrovert, Ambivert, with Introvert as the reference group) in logistic regression analysis*.

### Factors Predicting Adult Academic Outcomes in Boys With ADHD

#### Characteristics of Different Academic Groups at Follow-Up

Of the 101 follow-up boys with ADHD, only 32 (31.68%) entered senior middle school (at an average age of 15.54 ± 0.32 years), and the remaining 69 boys finished junior high school or studied in vocational secondary school. There were no differences in age at follow-up (19.35 ± 1.27 vs. 19.71 ± 1.22, *t* = 1.378, *p* = 0.171) or follow-up interval (9.41 ± 1.32 vs. 9.26 ± 1.07, *t* = 0.610, *p* = 0.543) between the higher and lower academic groups.

#### Comparisons of Childhood Characteristics Between Different Academic Groups

As shown in Table 3, we found that the higher academic level group had higher parental education levels than the lower academic level group (father: *t* = 5.924, *p* < 0.001; mother: *t* = 4.923, *p* < 0.001). The father's character varied significantly between the two academic level groups (χ^2^ = 6.299, *p* = 0.044), and the family economic level was higher in the higher academic level group than in the lower academic level group (*t* = 12.040, *p* < 0.001). There were no baseline significant differences in age at diagnosis, ADHD subtypes, incidence of oppositional defiant disorder, only child or not, character of the child and mother, maternal health during pregnancy, birth and delivery status, family history, and treatment or not between the higher and lower academic level groups. Regarding the baseline intelligence of the boys with ADHD, our results indicated that the higher academic level group had better baseline VIQ (*t* = 4.906, *p* < 0.001), PIQ (*t* = 3.065, *p* = 0.003), and FIQ (*t* = 5.393, *p* < 0.001) evaluated by the C-WISC compared to the lower academic level group.

**Table 3:** Comparisons of childhood characteristics between different academic groups.

	**Lower academic level group**	**Higher academic level group**	***t*/*X*^**2**^**	***P***
	***N* = 69**	***N* = 32**		
Age at diagnosis (year)	10.45 ± 1.36	9.93 ± 1.63	1.661	0.100
Education level				
Father	10.19 ± 2.61	13.66 ± 2.99	5.924	<0.001
Mother	10.03 ± 2.92	13.09 ± 2.83	4.953	<0.001
Family economic (High/Mid/low)	2/65/2	8/23/1	12.040	0.002
Treatment (Yes/No)	39/30	18/14	0.001	0.980
ADHD type (I/HI/C)	26/1/42	8/1/23	1.766	0.414
Character (Introvert/Extrovert/Ambivert)				
Father	14/33/22	13/8/11	6.229	0.044
Mother	7/38/24	1/24/7	3.961	0.138
Child	18/27/24	7/16/9	1.060	0.589
Only child (Yes/No)	52/17	18/14	3.754	0.053
Maternal health during pregnancy (Yes/No)	52/17	18/14	3.754	0.053
Delivery status (Normal natural labor/Cesarean section/Abnormal natural labor)	33/29/7	15/16/1	1.676	0.433
Birth status (health/Unhealth)	56/13	27/5	0.154	0.694
Oppositional defiant disorder (Yes/No)	8/61	4/28	0.017	0.896
Family history (Yes/No)	23/46	8/24	0.714	0.398
ADHD-RS-IV				
Inattention	16.46 ± 3.08	16.09 ± 3.37	0.545	0.587
Hyperactivity-impulsivity	11.25 ± 4.58	12.91 ± 5.39	1.600	0.113
CPRS-48				
Conduct problems	0.83 ± 0.48	0.91 ± 0.47	0.718	0.475
Learning problems	1.77 ± 0.60	1.57 ± 0.62	1.553	0.124
Psychosomatic problems	0.27 ± 0.30	0.26 ± 0.29	0.258	0.797
Impulsive-hyperactive index	1.27 ± 0.51	1.32 ± 0.69	0.060	0.671
Anxiety	0.46 ± 0.42	0.57 ± 0.42	1.260	0.211
ADHD index	1.21 ± 0.45	1.30 ± 0.49	0.872	0.385
IQ				
VIQ	99.44 ± 15.26	114.52 ± 12.18	4.096	<0.001
PIQ	96.75 ± 11.62	104.50 ± 12.27	3.065	0.003
FIQ	97.99 ± 12.00	111.02 ± 9.54	5.393	<0.001
FES-CV				
Cohesion	7.36 ± 1.53	7.22 ± 1.46	0.423	0.673
Expressiveness	5.47 ± 1.35	5.34 ± 1.53	0.422	0.674
Conflict	3.42 ± 1.93	3.45 ± 2.04	0.078	0.938
Independence	5.39 ± 1.36	5.38 ± 1.18	0.058	0.954
Achievement	6.66 ± 1.53	6.03 ± 1.90	1.778	0.078
Intellectual-cultural	3.75 ± 1.71	4.67 ± 1.97	2.391	0.019
Active-recreational	3.36 ± 1.94	4.23 ± 2.14	2.037	0.044
Moral-religious	5.27 ± 1.44	4.72 ± 1.43	1.788	0.077
Organization	6.36 ± 1.73	5.84 ± 2.31	1.240	0.218
Control	3.88 ± 1.92	3.11 ± 1.53	2.007	0.047

*Data were presented in Mean ± SD or N*.

In addition, the higher academic level group had a lower control index score (*t* = 2.007, *p* = 0.047) but a higher intellectual–cultural (*t* = 2.391, *p* = 0.019) and active–recreational index score (*t* = 2.037, *p* = 0.044) on the FES-CV at baseline compared to the lower academic level group. There were no differences in other indices on the FES-CV and ADHD-RS-IV between the two groups (all *p* > 0.05).

#### Risk Factors for Predicting Adult Academic Outcomes in Boys With ADHD

With the variables screened by group comparison as independent variables (*p* < 0.05), a stepwise multiple logistic regression analysis was used to determine the independent predictors for adult academic outcomes in boys with ADHD. Our results revealed that the father's education level (β = 0.329, Wald χ^2^ = 10.519, *p* = 0.001), family economic level (β = −1.517, Wald χ^2^ = 3.926, *p* = 0.048), and VIQ on the C-WISC (β = 0.067, Wald χ^2^ = 8.631, *p* = 0.003) could independently predict adult academic outcome (see Table 4). This model shows a good predictive effect on adult academic outcome, with an AUC of 0.870 (*p* < 0.001, 95% CI = 0.796–0.944). The corresponding sensitivity and specificity were 0.813 and 0.783, respectively (see Figure 1B).

**Table 4:** Results of the stepwise logistic regression analysis: independent predictors for adult academic outcome in ADHD boy.

						**95% CI for Exp (B)**	
	**B**	**SE**	**Wald**	**Sig**	**Exp. (B)**	**Lower**	**Upper**
Father's education level	0.329	0.101	10.519	0.001	1.389	1.139	1.694
Family economic level	−1.517	0.765	3.926	0.048	0.219	0.049	0.984
VIQ	0.067	0.023	8.631	0.003	1.070	1.023	1.119

## Discussion

The present study investigated childhood factors that predict the risk of symptomatic persistence and academic outcome of boys with ADHD into adulthood. In our follow-up investigation of 101 boys with ADHD, 65.35% continued to meet full or partial DSM-IV criteria for ADHD when they were adults, and only 31.68% of them entered senior high school. Our results indicated that the father's character, impulsive–hyperactive symptoms evaluated by the CPRS-48, and intellectual–cultural index in the family (evaluated by the FES-CV) could predict the persistence of ADHD in adulthood, with an AUC of 0.770 (*p* < 0.001, 95% CI = 0.678–0.863). The corresponding sensitivity and specificity were 0.743 and 0.727, respectively. Furthermore, we found that the father's education level, family economic level, and VIQ could independently predict adult academic outcome, with an AUC of 0.870 (*p* < 0.001, 95% CI = 0.796–0.944). The corresponding sensitivity and specificity were 0.813 and 0.783, respectively.

Family characteristics have a profound impact on the experiences and life trajectories of offspring, playing an important role in emotional and behavioral development during childhood (31). Thus, the family environment may be relevant for the course of various mental and psychological diseases and ultimately clinical outcomes, including major depressive disorder (32), trichotillomania, obsessive–compulsive disorder (33), and ADHD (23). Character profiles are correlated with individual differences in goals and values, which are based on learning and the perceptions of self and others (34). The character traits of parents, as an important family environment factor, exhibit a remarkable influence on children's emotional and behavioral development (35). Most previous studies have demonstrated the temperament and character profiles associated with ADHD and could be used to distinguish ADHD from comorbid disease (34, 36, 37). However, the relationship between the character traits of parents in children with ADHD and clinical or academic outcomes into adulthood remains relatively understudied. In the present study, our results showed that the father's character trait was significantly associated with adult clinical outcomes in boys with ADHD, but we did not find that the mother's character predicted adult clinical and academic outcomes in those boys. The preliminary findings highlight the important role of fathers in the development of boys with ADHD. In truth, parents provide both the rearing environment and genes to their children. Thus, the observed father–offspring association may be wholly or partially explained by genetic factors shared between the parent and child (38). Future studies are warranted to verify our results and to reveal the exact mechanism of the influence of parents' character on children with ADHD.

Parental education attainment and family economic level are also important family factors and were significantly associated with the growth of children. Low education and family income could influence material conditions, parenting skills, social development, or stress and thereby influence children's mental health and learning (39). Early studies supported that low parental education and family income may be associated with impaired clinical prognosis in children with ADHD (23, 40). However, our present study found no significant differences in parental education and family economic levels between the symptomatic persistence and relief groups, which was in line with some previous studies (41, 42). The contradictory results may be explained by the different age ranges at baseline and follow-up intervals, as the ages at follow-up in our present study and previous studies that are consistent with our results were all 15–30 years old (41, 42), but patients in other studies were followed at a younger age (23, 40). Hence, the above evidence suggests that income and parental education levels may predict ADHD symptomatology through childhood but not later in life. Interestingly, our present study found that the higher academic level group had better family income status and higher parental education levels than the lower academic level group; in particular, the father's education level and family income could significantly predict the adult academic outcome of boys with ADHD. A recent study supported our findings and demonstrated that parental education level and household income could influence not only adult education attainment but also occupational functioning, sexual behavior, and emotional functioning in children with ADHD (14). In addition, existing studies also provide evidence that supports that parental education and economic levels have a greater impact on child educational outcomes in non-ADHD individuals. A more recent study including 10,262 typically developing American children between ages 9 and 10 found that high parental education and household income contributed to children's whole-brain cortical surface area, which may be associated with brain development (43). Another study showed that school performance increased in youth when parental education was improved (44). Sociologists also found that wealthier families were frequently better able to provide offspring with superior resources, and these, in turn, assure their academic success (45). Therefore, through providing additional financial support to children with lower family economic incomes and encouraging parents to continue learning, management of those areas early on may assist in improving their adult functioning, including educational attainment. The mechanism underlying this intergenerational transmission of educational attainment requires further exploration but includes genetic influences and gene–environment interaction effects (45).

In the present study, we found that the intellectual–cultural and active–recreational indices on the FES-CV were associated with clinical and academic outcomes. Furthermore, the intellectual–cultural index could independently predict adult symptomatic persistence in boys with ADHD. Early literature indicated that a poor family atmosphere and function exacerbate the severity of ADHD and its prognosis (46, 47). Ample evidence further supports that parental training exerts considerable effects in reducing symptoms of ADHD and school performance (48, 49). However, different parenting training may cause varying effects. This may suggest that certain components of family relationships and function are more associated with ADHD. In the present study, a widely used tool, the FES-CV, was applied to evaluate family relationships and functions, which included 10 dimensions: cohesion, expressiveness, conflict, independence, achievement, intellectual–cultural, active–recreational, moral–religious, organization, and control. This is the first study to investigate family function factors to predict adult clinical and academic outcomes based on the FES-CV, and our preliminary findings suggest that intellectual–cultural and active–recreational factors may be the most important family functional components to predict adult outcomes in ADHD. Hence, early interventions to improve the family atmosphere by increasing intellectual–cultural and active–recreational activity may be beneficial to ADHD prognosis. Interestingly, there is also evidence suggesting that parents of children with ADHD had higher levels of parenting stress and home chaos and lower levels of parental efficacy than parents of children without ADHD (50). This means that raising a child with ADHD is likely to aggravate family stress and evoke poor parenting. Thus, the abovementioned evidence indicated that the relationship between family atmosphere and ADHD severity or prognosis is complex and merits future exploration.

Most previous studies demonstrated that higher IQ had protective effects on functional impairment in children with ADHD, including educational attainment, occupational rank, and social and occupational adjustment (12, 15). In the present study, we further found that IQ, especially VIQ, could significantly predict adult academic outcomes in ADHD. As no study to date has divided IQ into VIQ and PIQ, our findings need to be verified in the future. Interestingly, we did not find an association between childhood IQ and adult ADHD symptom persistence in the present study, which was consistent with some previous studies (14, 51) but inconsistent with other studies (10, 30). The discrepancy may have some explanations, such as the differences in sample size, age at IQ evaluation, follow-up interval, and definition of outcome (15). Even so, IQ in children with ADHD should be taken seriously, and more effective methods should be developed to improve general cognitive ability in these children.

Strong evidence supports that the severity of childhood ADHD symptoms as reported by parents was a strong predictor of ADHD outcome at follow-up (15, 30). Our results also indicated that the symptomatic persistence group had a higher impulsive–hyperactive subscale score and ADHD index on the CPRS-48 than the symptomatic relief group. Furthermore, the impulsive–hyperactive index was an independent and strong predictor of adult symptomatic persistence in boys with ADHD. Interestingly, we found that only the CPRS-48 but not the ADHD-RS-IV scale could be used to predict adult clinical outcomes in our ADHD sample. The CPRS-48 and ADHD-RS-IV are both widely used parent rating tools to evaluate ADHD symptoms but with different items and dimensions (26, 28). To the best of our knowledge, many tools have been developed in the field of mental health to assess clinical symptoms but usually have different reliability and validity in evaluating the clinical symptoms of the same disease (52). However, no study has compared the efficacy of the CPRS-48 and ADHD-RS-IV when evaluating ADHD symptoms, and future studies are warranted.

In the present study, we used parent-reported ADHD symptoms, rather than self-reported symptoms, which made our results more reliable because ample evidence supports that young individuals tend to report fewer symptoms and that self-reported ADHD symptoms were poorly differentiated by objective measures (15, 53). However, several limitations of this current study should be mentioned here. First, the size of the sample was relatively small, and a large proportion of participants from the original cohort were missing from the adult assessments, which may have influenced the findings to some extent. Second, only male but not female patients were included in the present study, which limited the conclusions to male patients only. Third, only limited factors were explored in the present study. Other important factors, including treatment methods and duration, educational environment, and school level, were not investigated and were hard to control but might have great impacts on the outcomes. Fourth, demographic and clinical data collected by the self-designed questionnaire might have been somewhat subjective, although professional guidance was given. Fifth, the participants were all in early adulthood at follow-up, and some of them may have remitted at a later date, which may have influenced our conclusion. Therefore, future long-term follow-up investigations with larger samples, rigorous psychological experiments, or clinical designs to measure and control confounding factors are warranted to verify our findings.

In summary, our cohort study indicated that only 34.65% of boys with ADHD achieved symptomatic relief in early adulthood, and only 31.68% of them entered senior middle school. More importantly, we demonstrated the predictive value of the father's character trait and education level, children's VIQ, and impulsive–hyperactive as well as family economic level and intellectual–cultural function on adult clinical and academic outcomes in boys with ADHD. Although there is a long journey in clinical practice to develop appropriate interventions and improve the prognosis of children with ADHD, our finding represents an incremental increase in this knowledge to some extent.

## Data Availability Statement

The raw data supporting the conclusions of this article will be made available by the authors, without undue reservation.

## Ethics Statement

The studies involving human participants were reviewed and approved by The Third Affiliated Hospital of Soochow University. Written informed consent to participate in this study was provided by the participants' legal guardian/next of kin.

## Author Contributions

YR, HF, and XK conceptualized and designed the study. YR, GP, JC, and SW diagnosed patients and completed the screening assessments. XF and YR analyzed the data, performed the statistical analysis, and wrote the first draft of the manuscript. All authors contributed to the article and approved the submitted version.

## Conflict of Interest

The authors declare that the research was conducted in the absence of any commercial or financial relationships that could be construed as a potential conflict of interest.

## Publisher's Note

All claims expressed in this article are solely those of the authors and do not necessarily represent those of their affiliated organizations, or those of the publisher, the editors and the reviewers. Any product that may be evaluated in this article, or claim that may be made by its manufacturer, is not guaranteed or endorsed by the publisher.
