# Supplementary material for: Predicting the Adult Clinical and Academic Outcomes in Boys With ADHD: A 7- to 10-Year Follow-Up Study in China
Source: Front Pediatr. 2021 Aug 2;9:634633. doi: 10.3389/fped.2021.634633 (PMC8367416; doi:10.3389/fped.2021.634633)
Supplement: Supplementary file 1 [file Data_Sheet_1.zip › New Supplementary Material/Table S1.docx]

Table S1. Comparisons of baseline characteristics between follow-up and drop-out group

|  | Follow-up group  N=101 | Drop-out group  N=53 | t/t′/X^2^ | *P* |
| --- | --- | --- | --- | --- |
| Age at diagnosis (year) | 10.29±1.46 | 9.99±1.27 | 1.253 | 0.212 |
| Education level |  |  |  |  |
| Father | 11.28±3.17 | 10.66±3.2.99 | 1.189 | 0.236 |
| Mother | 11.00±3.22 | 10.34±2.57 | 1.293 | 0.198 |
| Family economic (High/Mid/low) | 10/88/3 | 6/44/3 | 0.782 | 0.677 |
| Treatment (Yes/No) | 57/44 | 28/25 | 0.183 | 0.669 |
| ADHD type (I/HI/C) | 34/2/65 | 16/0/37 | 1.335 | 0.513 |
| Character (Introvert/Extrovert/ Ambivert) |  |  |  |  |
| Father | 27/41/33 | 13/29/11 | 3.318 | 0.190 |
| Mother | 8/62/31 | 7/34/12 | 1.847 | 0.397 |
| Child | 25/43/33 | 18/21/14 | 1.575 | 0.455 |
| Only child (Yes/No) | 87/14 | 48/5 | 0.265 | 0.606 |
| Maternal health during pregnancy (Yes/No) | 70/31 | 33/20 | 0.778 | 0.378 |
| Delivery status (Normal natural labor /Cesarean section/Abnormal natural labor) | 48/45/8 | 26/25/2 | 0.990 | 0.610 |
| Birth status (health/Unhealth) | 83/18 | 46/7 | 0.461 | 0.544 |
| Oppositional defiant disorder (Yes/No) | 12/89 | 9/44 | 0.768 | 0.381 |
| Family history (Yes/No) | 31/70 | 33/20 | 0.778 | 0.378 |
| ADHD‐RS‐IV |  |  |  |  |
| Inattention | 16.35±3.16 | 15.79±3.82 | 0.960 | 0.339 |
| Hyperactivity-impulsivity | 11.77±4.89 | 11.81±5.31 | 0.046 | 0.964 |
| CPRS-48 |  |  |  |  |
| Conduct problems | 0.86±0.48 | 0.89±0.51 | 0.386 | 0.700 |
| Learning problems | 1.70±0.61 | 1.71±0.59 | 0.026 | 0.979 |
| Psychosomatic problems | 0.27±0.29 | 0.24±0.36 | 0.425 | 0.671 |
| Impulsive-hyperactive index | 1.28±0.57 | 1.33±0.64 | 0.498 | 0.619 |
| Anxiety | 0.49±0.42 | 0.55±0.39 | 0.783 | 0.435 |
| ADHD index | 1.24±0.46 | 1.29±0.55 | 0.587 | 0.558 |
| IQ  VIQ  PIQ  FIQ | 102.09±14.89  99.14±13.12  100.83±13.09 | 108.23±17.26  99.31±10.82  104.54±11.98 | 1.865  0.066  1.395 | 0.065  0.948  0.166 |
| VIQ | 104.22±15.94 | 101.19±12.14 | 1.211 | 0.228 |
| PIQ | 99.20±12.31 | 97.76±13.17 | 0.580 | 0.563 |
| FIQ | 102.12±12.78 | 99.76±12.01 | 1.113 | 0.267 |
| FES-CV |  |  |  |  |
| Cohesion | 7.31±1.50 | 7.13±1.96 | 0.651 | 0.516 |
| Expressiveness | 5.43±1.40 | 5.16±1.63 | 1.085 | 0.280 |
| Conflict | 3.43±1.96 | 3.45±2.04 | 0.070 | 0.944 |
| Independence | 5.39±1.30 | 5.55±1.22 | 0.755 | 0.452 |
| Achievement^a^ | 6.46±1.67 | 6.46±1.37 | 0.014 | 0.989 |
| Intellectual-cultural | 4.04±1.84 | 3.57±1.55 | 1.608 | 0.110 |
| Active-recreational | 3.64±2.03 | 3.57±1.55 | 0.204 | 0.839 |
| Moral-religious | 5.09±1.45 | 5.15±1.47 | 0.214 | 0.831 |
| Organization | 6.19±1.93 | 6.57±1.58 | 1.224 | 0.223 |
| Control | 3.64±1.83 | 4.32±1.98 | 2.141 | 0.034 |

Data were presented in Mean ± SD or N. ^a^ Only Achievement index in FES-CV showed homoscedasticity uneven, and t′-test were used to compare the difference in Achievement index between the follow-up and drop-out groups.
