# Supplementary figures and images for: Predicting the Adult Clinical and Academic Outcomes in Boys With ADHD: A 7- to 10-Year Follow-Up Study in China
Source: Front Pediatr. 2021 Aug 2;9:634633. doi: 10.3389/fped.2021.634633 (PMC8367416; doi:10.3389/fped.2021.634633)

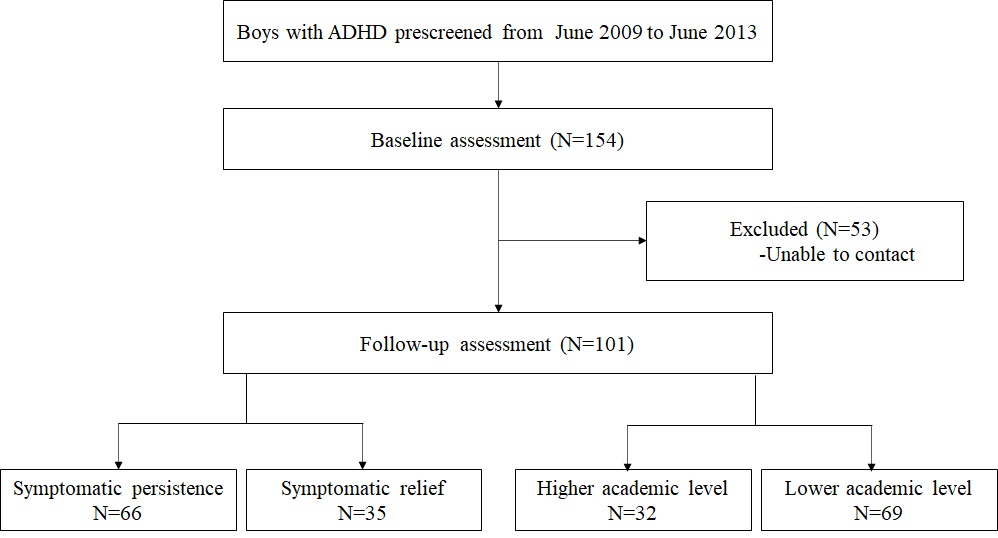

Supplement: Supplementary file 1 [file Data_Sheet_1.zip › New Supplementary Material/Figure S1.tiff]
